# Various Analytical Techniques Reveal the Presence of Damaged Organic Remains in a Neolithic Adhesive Collected During Archeological Excavations in Cantagrilli (Florence Area, Italy)

**DOI:** 10.3390/molecules31020274

**Published:** 2026-01-13

**Authors:** Federica Valentini, Lucia Sarti, Fabio Martini, Pasquino Pallecchi, Ivo Allegrini, Irene Angela Colasanti, Camilla Zaratti, Andrea Macchia, Angelo Gismondi, Alessia D’Agostino, Antonella Canini, Anna Neri

**Affiliations:** 1 Sciences and Chemical Technologies Department, Tor Vergata University, Via della Ricerca Scientifica 1, 00133 Rome, Italy; ireneangela.colasanti@students.uniroma2.eu (I.A.C.); camilla.zaratti@students.uniroma2.eu (C.Z.); 2 Dipartimento Scienze Storiche e Beni Culturali, University of Siena, Via Roma 53, 53100 Siena, Italy; lucia.sarti@unisi.it; 3 Museo e Istituto Fiorentino di Preistoria, Via dell’oriolo 24, 50122 Firenze, Italy; fabio.martini@unifi.it (F.M.); p.pallecchi@museofiorentinopreistoria.it (P.P.); 4 Envint Srl, Via Paradiso 65 a, Montopoli di Sabina, 02034 Rieti, Italy; ivo.allegrini@tiscali.it; 5 YOCOCU APS, Via Torquato Tasso 108, 00185 Roma, Italy; aps@yococu.com; 6 Laboratory of Archaeobotany DAPHNE (Diet, Ancient DNA, Plant-Human Nexus, and Environment), Department of Biology, Tor Vergata University, Via della Ricerca Scientifica 1, 00133 Rome, Italy; gismondi@scienze.uniroma2.it (A.G.); d.agostino@scienze.uniroma2.it (A.D.); canini@uniroma2.it (A.C.); 7 Department of Biomedicine and Prevention, Tor Vergata University, Viale Montpellier 1, 00133 Rome, Italy; anna.neri@uniroma2.it

**Keywords:** triterpenes, beeswax, benzoyl resins, gelatinized starch, gas chromatography–mass spectrometry, infrared spectroscopy, nuclear magnetic resonance spectrometry, archeobotany

## Abstract

In this work, an archeological adhesive collected at Cantagrilli (near Florence) was chemically analyzed by applying gas chromatography/mass spectrometry, infrared spectroscopy, and nuclear magnetic resonance spectrometry combined with the archeobotanical investigations. Data identify triterpenes, aged anhydride, benzoyl resin, and gelatinized starch in the sample. The multi-analytical approach allowed us to identify some molecular compounds, as well as their state of chemical decomposition (especially by applying the mass spectrometry techniques). On the other hand, archeobotanical measurements have provided useful but not unequivocal information regarding the possible origin of triterpenes from some terrestrial plants, combined with the presence of microorganisms and transformed chemicals (such as starch modified into gelatin). All this information is very useful to Prehistoric Archeologists for understanding the cultural processes and technologies used by ancient populations.

## 1. Introduction

**Figure 1:**
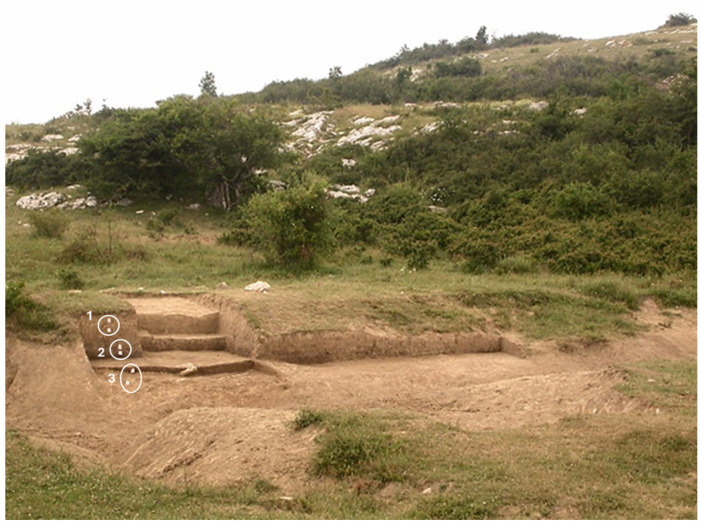
The excavation site at Cantagrilli, with the (1, 2, 3) areas of investigation.

The use of natural products with strong adhesive properties, widely applied in the past to assemble tools, which has been extensively documented in the literature [1,2], has pushed researchers and enthusiasts of Prehistoric Archeology to characterize the properties of these natural substances/compounds to better understand the manufacturing processes that prehistoric man adopted in his daily life. Often, the procurement and subsequent processing of natural adhesives take place according to procedures that depend on the geographical origin, therefore on the raw materials contained therein and on the processing customs of the populations settled there [3,4,5]. A wide range of materials exhibit natural adhesive properties, such as fresh tree resin, but these adhesives often need to be further processed. According to the current state of the art [5], three main processes can be distinguished: (i) chemical reactions (e.g., two-component adhesives such as cement), (ii) distillation or pyrolysis of biomass (e.g., birch bark and pine tar), (iii) hydrolysis of animal matter (e.g., hide glue). Sometimes, the base material requires further improvement, because it is either too brittle or too soft for the intended function. In such a case, additives like ochre and beeswax may be added to manipulate the adhesive’s material properties. In particular, natural resins (which are exudates secreted by plants and insects generally for protection) have been widely applied as adhesives for tool assembling. Resins are water-insoluble compounds and consist of hydrocarbons like terpenoids, toluene, and waxes [6,7]. When Fourier-Transform InfraRed spectroscopy (FTIR) is used as a characterization technique, resins can be identified into broad groups based on terpenoids and phenolic compounds (for plant resins) and polyesters and waxes (for insect resins). In particular, the latter (i.e., insect resins or lacs) are excreted by insect from the Kerriidae family as they forage the branches of trees. Insect resins often were used as a varnish on wooden objects, gildings, and paintings in historic Europe and eastern Asia [7,8]. Resins that derive from plants (i.e., those of vegetal origin) are secreted by plants to protect themselves from herbivores, dehydration, pathogens, and environmental damage caused by events such as fires and storms [7,9,10]. Plant resins consist of volatile and non-volatile compounds as terpenoids, including resin acids, and/or phenolic compounds that are lipid-soluble and water-insoluble as well as other, sometimes characteristic, compounds like toluene in spinifex resin [6,7,11]. Many plants produce resins, archeologically the most notable ones are conifers of the Pinaceae and deciduous trees of the Burseraceae families. Some European-specific examples are Scots pine (*Pinus sylvestris*), Norway spruce (*Picea abies*), balsam fir (*Abies balsamea*), European larch (*Larix decidua*). Resins are perhaps the most widespread adhesives in prehistory; 64,000 years ago, *Podocarpus* sp. resin, sometimes loaded with ochre and possibly quartz and burnt bone, was used to haft stone tools in South Africa [12,13]. Neanderthals [14] and European Mesolithic hunter-gatherers [15] used resins to haft stone tools. In addition, European Iron Age people used resin to waterproof and repair pots [16]. According to these considerations, there are three main specific groups for plant resins, i.e., diterpenoids, triterpenoids, and aromatic esters/phenolics, investigated by ATR/FTIR (Attenuated Total Reflection/Fourier-Transform Infrared) spectroscopy, according to the literature [17]. GC-MS (gas chromatography–mass spectrometry) can be more specific regarding species, production methods, and stages of refinement [7,18]. Before application, resins are softened by heat or dissolved in oils. Extensive heating removes volatiles and hardens the material. Sometimes, resins then are mixed with plasticizers like beeswax and animal fat to make them less brittle. These resins and ambers also appear to have contained microorganisms accidentally trapped in these viscous media of higher plants [19]. The site of Cantagrilli (Figure 1) is located on the southern summit of the Calvana mountains, a pre-Apennine ridge composed mainly of limestone rocks of the geological formation of Monte Morello (Paleogene–Middle Eocene). The ridge is characterized by a rounded subhorizontal crest and steep sides with a maximum height of 916 m a.s.l. It has a typically karst morphology, with the presence of caves, numerous sinkholes, resurgences, and swallow holes. This peculiarity supports a particular flora typical of calcareous soils where there are numerous endemisms. The Early Neolithic finds were found in the summit esplanades in the Southern part of the ridge at a height of about 780 m in correspondence with the partially washed surface of a sinkhole. The analyzed find was part of a partially washed away archeological deposit including flint artifacts and some ceramic fragments [19].

In this work, a combination of GC-MS (gas chromatography–mass spectrometry), FTIR (Fourier-Transform Infrared spectroscopy), and ^1^H proton and ^13^C carbon-NMR (Nuclear Magnetic Resonance Spectrometry) techniques and also the archeobotanical investigations (these latter, mainly performed by Optical Microscopy) highlight the natural materials and their state of chemical decomposition, in which they have reached us, at the current state of study. The molecular findings are certainly interesting because they offer Prehistoric Archeology experts new food for thought regarding the processes and technologies used to transform materials into lithic tools, useful for hunting and everyday life. The multi-analytical approach is currently the most robust in offering abundant scientific information useful in many fields, including Archeology, which can then better understand aspects regarding cultures, technologies, and transformations of the past.

## 2. Results

In this section, all experimental results obtained by GC-MS, FTIR, NMR, and archeobotanical analyses are described. The scheme and order used to describe the results and their discussion are also described in the experimental section, presenting the analytical techniques in the same order as the experimental data.

### 2.1. GC-MS Experimental Results

**Figure 2:**
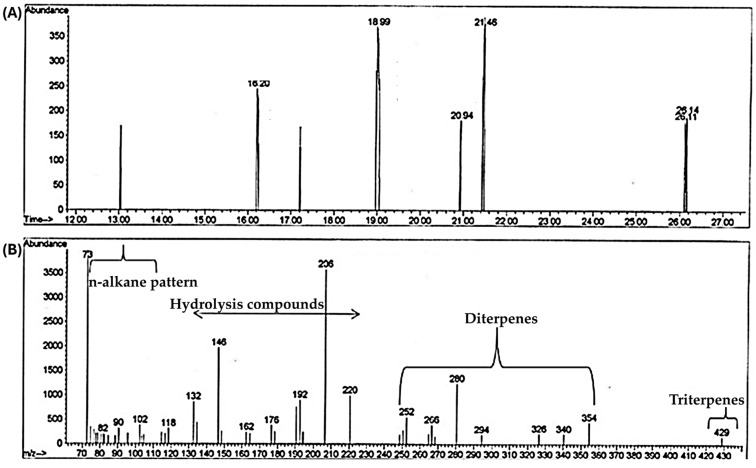
(**A**). Total Ion Chromatogram (TIC) of the extracted samples. (**B**). Mass spectrum where the families of the main compounds recorded have been identified: triterpenes, diterpenes, hydrolysis products, and n-alkane pattern (shown, in more detail in Table 1).

In this paragraph, the main results confirm what was demonstrated by XRD in a previous work [19]. Especially Figure 2, mainly reveals the presence of terpenoids (primarily triterpenes and diterpenes), hydrolysis products, and n-alkane patterns, respectively. These investigations represent an important contribution to the understanding of prehistoric technologies and material processing.

**Figure 3:**
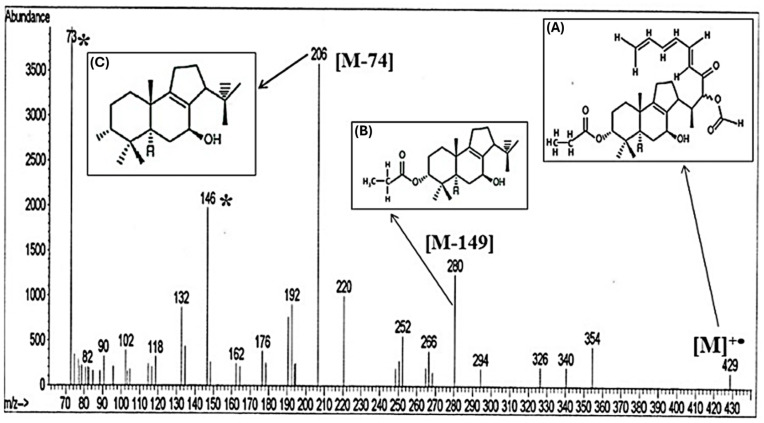
Mass Spectrum of molecules and also their corresponding trimethylsilyl derivatives (marked with *). A Class: acyclic diterpene; Pathway: terpenoids; Super Class: sesterterpenoids. B Class: acyclic monoterpenoids; Pathway: terpenoids; Super Class: sesquiterpenoids. C Class: monoterpenes; Pathway: terpenoids; Super Class: monoterpenoids.

Figure 3 shows the mass spectrum of the samples, where all the main fragments, typical of the molecular fragmentation related to the residual organic component found in the analyzed sample, have been also indexed and attributed (see also Table 2).

### 2.2. FTIR Results

**Figure 4:**
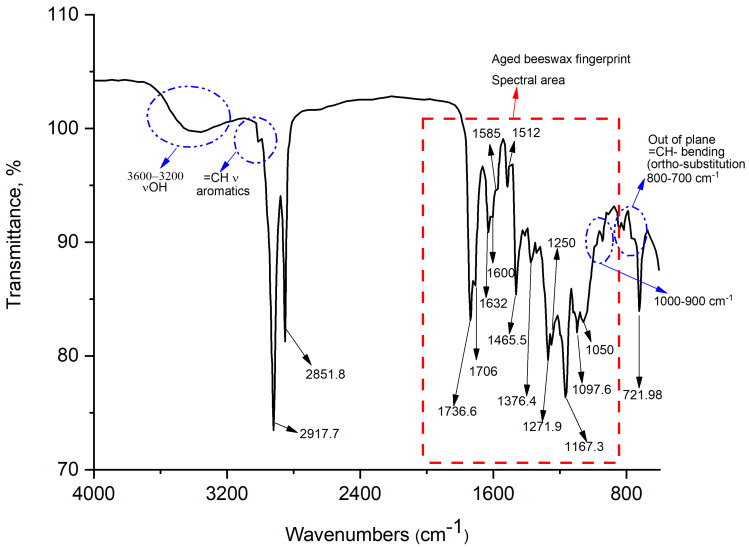
Typical FTIR spectrum with assignment of highlighted absorption bands. The red area shows a typical FTIR fingerprint exhibited by aged beeswax.

The FTIR data (Figure 4, Table 3) highlight the beeswax spectral fingerprint, in agreement with what was found in the XRD study of the same sample, published in a previous article [19]. Especially, two intense bands recorded at about 2917–2920 and 2848–2851 cm^−1^ can be assigned to the asymmetric and symmetric stretching vibrations (ν_as_; ν_s_) of methylene (-CH_2_-) and methyl groups (-CH_3_), respectively. The latter functional groups could certainly be related to the hydrocarbon part of the apolar tail of beeswax, also in agreement with what is reported in the literature [20]. The =C-H stretch in aromatics is observed at 3100–3000 cm^−1^. Note that this band occurs at a slightly higher frequency than the C-H stretch in alkanes. Aromatic hydrocarbons show absorptions in the regions 1600–1585 cm^−1^ and 1500–1400 cm^−1^ due to carbon–carbon stretching vibrations in the aromatic ring (as also summarized in Table 3). Bands in the region 1250–1000 cm^−1^ are due to C-H in-plane bending, although these bands have a weak signal intensity. The pattern of the out-of-plane C-H bending bands in the region 900–675 cm^−1^ is also characteristic of the aromatic substitution pattern (especially, the spectral region ranging from 700 to 800 cm^−1^ is typical of the ortho substitution in the aromatic ring; while the spectral range between 800 and 900 cm^−1^ concerns a para substitution in the aromatic ring). The pattern of overtone bands in the region 2000–1665 cm^−1^ reflects the substitution pattern on the ring, but in this case of study these bands are very weak in intensity, and, for this purpose, reference is made only to the bands recorded at low frequencies (i.e., those attributable to the bending vibration modes of chemical bonds; see Figure 4 and Table 3).

In addition, the adsorption band centered at about 1703 cm^−1^ has been also recorded, probably due to the stretch of C=O bond, typical of the ester groups. The signal recorded at 1700 cm^−1^ could indicate the presence of highly esterified carboxyl groups of fatty acids in the aged beeswax sample (according to the XRD results reported in a previous study) [19]. These two bands (centered at 1703 and 1700 cm^−1^, Tautomer Markers) could also represent a typical coupling signal between two carbonyl functional groups, belonging to the anhydride molecules, as happens in the keto–enol tautomerism [20]. Furthermore, the absence of the adsorption band centered at 1800 cm^−1^ (typical of anhydrides) could confirm a possible coupling between two carbonyl groups, which are not always exactly equivalent by symmetry, displaying coupling to each other (keto tautomer), as described by Silverstein et al. (2005) [20]. Moreover, coupling is not an exclusive phenomenon of anhydrides but also of many other functional groups in organic chemistry, such as many acyl derivatives including esters.

In this case, coupling signals related to keto–enol tautomerism could also be observed in the absorption spectrum at 1632 cm^−1^ (C=O hydrogen bonded) and 2400–3200 cm^−1^ (O-H hydrogen bonded), respectively, as highlighted in Figure 4, (Tautomer Markers). The presence of anhydrides and esters is also confirmed by the signal recorded at 1050 cm^−1^, typical of the simple C-O bond that is contained in the anhydride and ester molecules, respectively [20].

**Table 3:** FTIR molecular adsorption band assignments for the sample.

Wavenumber (cm^−1^)	Vibration Mode	Functional Groups	References	Notes
3200–3500	Stretching vibrations	OH hydrogen bonded	[20]	Phenolic resins [21,22]
3000–3100	Stretching vibrations	=C-H	[20]	Phenolic resins [21,22]
2917–2920	Stretching vibrations (ν_as_)	-CH_2_-	[23]	Phenolic resins [21,22]
2848–2851	Stretching vibrations (ν_s_)	-CH_3_	[20]	Phenolic resins [21,22]
1694–1703	(ν_as_ out-of-plane)	-C(=O) in esters group	[20]	Aged beeswax [23,24]
1700	(ν_s_) anhydride	C(=O)-O-C(=O)	[25]	Aged beeswax [23,24]
1703	(ν_as_) anhydride	C(=O)-O-C(=O)	[25]	Aged beeswax [23,24]
1632	Asymmetric stretching vibrations (ν_as_)	C(=O) hydrogen bonded	[20]	Aged beeswax [23,24] Vanillin [24]
1585–1600	ν in aromatic ring	-C=C-	[20]	Aged beeswax [23,24] Phenolic resins [21,22]
1400–1500	ν in aromatic ring	-C=C-	[20]	Aged beeswax [23,24] Phenolic resins [21,22]
1465	δ_scissoring_ hydrocarbon chain	CH_3_-(CH_2_)_n_-CH_3_	[20]	Aged beeswax [23,24] Phenolic resins [21,22]
1376–1327	δ (CH_3_) ν/δ C(=O)-OH	Methyl (CH_3_) bending stretching/bending combination	[26]	Phenolic resins [21,22] Aged beeswax [23,24]
1000–1250	In-plane bending δ	-CH-	[20]	Phenolic resins [21,22]
1167	ν	C(=O)O- esters	[20]	Aged beeswax [23,24]
1150	δ ether groups in pure vanillin	-CH- in ester chain	[25]	Phenolic resins [21,22] Vanillin [24]
1050	ν ethers in esters and anhydride groups	C(=O)-O- C(=O)-O-C(=O)	[25]	Aged beeswax [23,24]
800–900	Para substitution in the aromatic ring	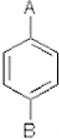 1,4	[20,21]	Phenolic resins [21,22]
700–800	Orto substitution in the aromatic ring	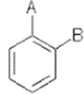 1,2	[20,21]	Phenolic resins [21,22]
721.98	δ long aliphatic chains	CH_3_-(CH_2_)_n_-CH_3_	[20,21]	Phenolic resins [21,22]

Peaks falling in the region characteristic of aromatic C=C bonds (1600–1450 cm^−1^) and in the deformations of aromatic C-H bonds (1000–650 cm^−1^) are characteristic of benzoyl resin.

### 2.3. NMR Results

**Figure 5:**
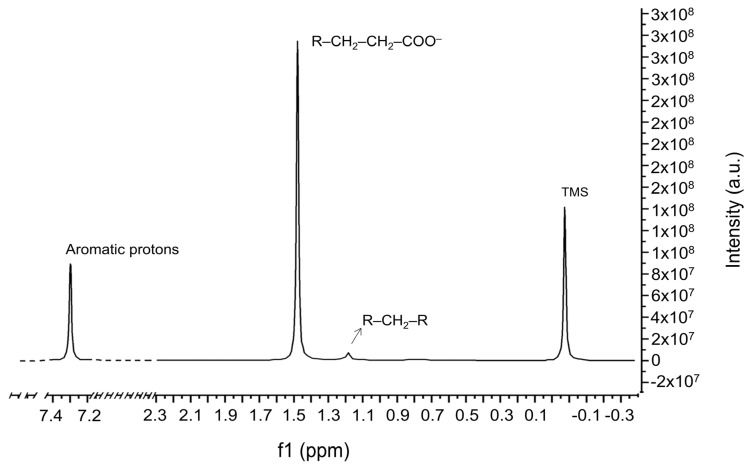
The ^1^H-NMR spectrum of the sample.

NMR spectrometry is complementary to the GC-MS and FTIR techniques reported above. The ^1^H-NMR spectrum (shown in Figure 5) highlights a typical fingerprint of all the nuclei examined, such as esters, which represent the polar component of oils, such as esters of fatty acids, methylene functional groups as components of the non- polar tails of a hydrocarbon nature of the oils, [24] and the aromatic protons of flavonoids, according to the literature [22,27]. All these detailed assignments have been also shown in Table 4.

A solid-state ^13^C NMR spectrum was collected for the sample; it mainly indicates long alkyl chain lengths (probably related to the presence of the methyl palmitate compound) and the packing of the alkane chains in the solid wax, giving an extra intermolecular Van der Waals force, which is not observed in the solution state. An additional peak is observed at 53 ppm, which could be assigned to oxygenated functional groups, mainly C-OH, C-O, and C(=O).

Especially, these latter are probably attributable to the ester functional groups, in accordance with what is reported above in the text and in the literature [24], and for this purpose, this signal is always coupled to that recorded around 174.26 ppm, typical of ester carbon functional groups (-C(=O)O).

In addition, there are some peaks assigned to the α-carbon recorded at 14–16 ppm, the β-carbon at 24–26 ppm, and the other carbon atoms (γ, δ, and ε), the latter probably assigned to a complex peak or group of peaks, detected at 28–38 ppm (see also Table 4). These results agree with those reported by other authors in the literature [28,29] and, for that matter, with the shorter-chain alkanes in solution-state ^13^C NMR. The peaks around 131.88 and 127.08 ppm indicated the unsaturation in methyl esters (-C=C-; Csp^2^, as highlighted in Table 4).

**Table 4:** ^1^H-NMR and ^13^C-NMR spectral data and chemical shift assignments.

^1^H-NMR Spectral Data and Chemical Shift Assignments			
Chemical Functional Groups	Description	^1^H-NMR (ppm)	References
R-CH_2_-R	Methylene group	1.29	[24]
R-CH_2_-CH_2_-COO^−^	Ester-carboxylic acid	1.62	[24]
Protons of flavones	Aromatic protons of flavones AA’BB’ systems	7.30	[22,30]
**^13^** **C-NMR Spectral Data and Chemical Shift Assignments**			
**Chemical Functional Groups**	**Description**	**^13^** **C-NMR (ppm)**	**References**
α-CH_3_	α-Carbon of chain alkanes	14–16	[28,29]
β-CH_2_	β-Carbon of chain alkanes	24–26	[28,29]
γ-CH_2_; δ-CH_2_; ε-CH_2_	γ, δ, and ε-Carbon of chain alkanes	23–38	[28,29]
C-OH; C-O	Oxygenated species (ethers; alcohols)	53	[28,29]
C(=O)O-	Oxygenated species (mainly esters)	174.26	[24]
(-C=C-; Csp^2^)	The unsaturation in methyl esters	131.88 and 127.08	[31]

**Figure 6:**
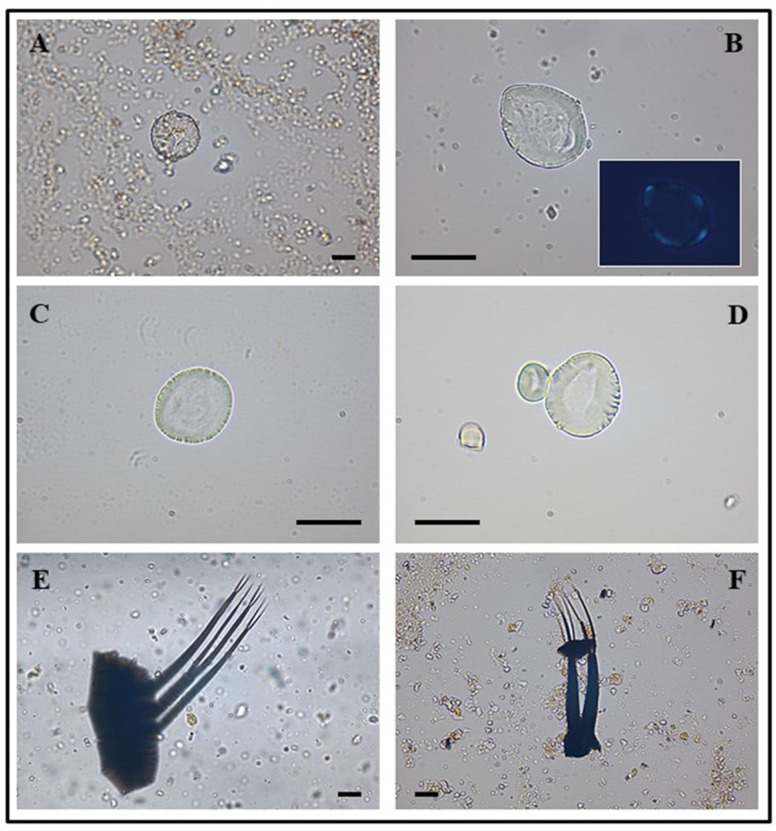
Archeobotanical microdebris and images captured by optic microscopy are shown. *Cupressaceae* ancient pollen (**A**); Hordeeae ancient starch (**B**); reference starch from *Hordeum vulgare* (**C**); reference starch from *Quercus pubescens* (**D**); microdebris of doubtful origin (**E**,**F**). The scale bar indicates 15 μm.

The archeobotanical investigations is shown in Figure 6. Overall, microscopic research disclosed the presence of one pollen type and one starch morphotype in the studied sample, which will be discussed in Section 4. The non-pollen palynomorph (NPP) group included several fragments of microarthropods (see also Figure 6).

## 3. Discussion

The main chemical components identified in this sample (Scheme S1) are as follows: terpenes; aged beeswax (and not bee honey); benzoyl resin and gelatinized starch. As for terpenes, in the experimental conditions, which consist of extraction, sample derivatization, and analysis in GC-MS according to the literature [32,33], the extract was mainly made up of oxygenated monoterpenes, with 3-methyl-4-(2,6,6-trimethyl-2-cyclohex-en-1-yl)-3-buten-2-one as the most abundant compound (6383 ng/mL), followed by 12(*S*)-hydroxy-(5*Z*,8*E*,10*E*)-heptadecatrienoic acid (2044 ng/mL) and 4-[[(8*R*,9*R*,10*R*,11*R*,13*S*,14*R*,17*S*)-17-acetyl-10,13-dimethyl-3 oxo1,2,6,7,8,9,11,12,14,15,16,17-dodecahydrocyclopenta[a]phenanthren-11-yl]oxy]-4-oxobutanoate (64 ng/mL).

The analysis was carried out using a calibrated terpenes standard mixture, allowing the semi-quantitative determination of three organic compounds (see Table S1 in Supporting Information). This distribution allows us to state that oxygenated monoterpenes are the most abundant fraction in the sample, followed by diterpenes, and lastly (in terms of concentration) sesterterpenes. The analysis was performed on a single, very well-characterized extract, providing a representative chemical fingerprint under the tested experimental conditions. Although the lack of replicates makes statistical data processing inaccurate, the dataset offers a consistent reference for subsequent applications using the same procedures.

The full dataset, including Retention Times, NIST molecular formulas, NIST exact and theoretical masses, and Peak Rating (Max.) match scores, is reported in Table 5. In some cases, molecular compounds were detected at multiple Retention Times but shared the same molecular formula and theoretical mass. These signals, retained in the final Table 5, could represent isomers, but this discussion firstly focuses on the most relevant compounds, the five molecules with the highest peak areas, each with a Total Score > 95 and a Peak Rating ≥ 9.5.

In particular, the C_25_H_33_O_6_ compound (belonging to the sesterterpenes-C25, Scheme S2) was detected as the molecular ion peak recorded at values of *m*/*z* = 429, that corresponds to the protonated radical cation [M−H]^+•^, (see Figure 3 and Table 2). Subsequently, this compound loses a fragment [M−75], generating the peak recorded at 354 *m*/*z*. The fragment *m*/*z* = 75 corresponds to the loss of a functional group such as the ester compound, which, in the structure of the terpenoid at *m*/*z* = 429, is found in the lateral branching of the identified molecule (see Scheme S2). From the peak at *m*/*z* = 354, a series of low-intensity peaks are observed, obtained mainly from the fragmentation and subsequent loss of methylene groups (CH_2_, *m*/*z* = 14). In the molecular fragmentation involving the peak at *m*/*z* = 326 and generating the peak at *m*/*z* = 294, the lost fragment is equal to 32 *m*/*z* (indicative of functional alcohol groups). The sum of all these lost fragments (3 × CH_2_; one esther group and one alcohol group) leads to the overall mass loss corresponding to [M-149], which generates the medium-/low-intensity peak centered at *m*/*z* = 280 (C_17_H_28_O_3_), in the mass spectrum (Figure 3 and Table 2). The lost mass fragment of 149 corresponds precisely to the lateral branching of the identified terpenoid (i.e., C_25_H_33_O_6_). That branching containing the oxygen functionalities is not chemically stable (probably explained by the damage processes associated with exposure to light, air, and environmental microorganisms) and rearranges itself by a typical [4+2] Diels–Alder reaction mechanisms [34], thus losing a molecular fragment coinciding with phthalic anhydride (see Scheme S3). Bonaduce et al., 2017 [35], state that phthalic acid or phthalic anhydride can also be present in the alkyd resin as a terminal chain or length modifier. Especially, phthalic anhydride can be detected in the pyrogram of an alkyd archeological resin without a derivatizing agent [36].

Then, the peak detected at *m*/*z* = 280 loses the molecular fragment [M−74] (which corresponds to esters and/or carboxylic acids, contained in archeological lacquers [37] and also in oily substances [38]) generating the basic peak recorded at the value of *m*/*z* = 206 (i.e., C_14_H_22_O, Mol. Mass, Retention Time according to the standard reported in Table 5, with a Peak Rating of 10).

The peaks recorded at *m*/*z* of 73 * and 146 * are assigned to trimethylsilyl derivatives [39], according to the analysis of the standards (trimethylsilyl radical C_3_H_9_Si *m*/*z* = 73.18 *; 3-trimethylsilyl propionic acid C_6_H_14_O_2_Si *m*/*z* = 146.25 *) reported in Table 5, where all the NIST parameters, the Retention Time and the Peak Rating (Max.) are perfectly coincident.

**Table 5:** The identified compounds by targeted GC-MS. For each compound, the Retention Time (RT), molecular formula (as matched with NIST library), and theoretical and observed molecular masses are reported. The Peak Rating column indicates the library match quality on a scale from 1 to 10.

Name	NIST Formula	NIST Observed Mol. Mass	NIST Theo. Mol. Mass.	Rt (min)	Peak Rating (Max.)	Notes and Examples
Trimethylsilyl radical	C_3_H_9_Si	73.18 *	73.19	17.19	10	A typical silyl derivative radical involved in the molecular fragmentation mechanism of fats, included into archeological adhesives, prehistoric resins, and waxes [40,41]
3-(Trimethylsilyl) propionic acid	C_6_H_14_O_2_Si	146.25 *	146.26	18.99	10	A typical silyl derivative radical involved in the molecular fragmentation mechanism of fats, included into archeological adhesives, prehistoric resins, and waxes [40,41]
3-Methyl-4-(2,6,6-trimethyl-2-cyclohexen-1-yl)-3-buten-2-one	C_14_H_22_O	206.31	206.32	20.94	10	Sesquiterpenoids contained in the resins of *T. mucronatum* [42]
12(*S*)-Hydroxy-(5*Z*,8*E*,10*E*)-heptadecatrienoic acid	C_17_H_28_O_3_	280.38	280.40	21.46	9.8	*Calocedrus formosana Florin* (sesquiterpene family); essential oils and extractives of coniferous tree, classified in the family of *Cupressaceae* [43]
4-[[(8*R*,9*R*,10*R*,11*R*,13*S*,14*R*,17*R*)-17-acetyl-10,13-dimethyl-3-oxo-1,2,6,7,8,9,11,12,14,15,16,17-dodecahydrocyclopenta[a]phenanthren-11-yl]oxy]-4-oxobutanoate	C_25_H_33_O_6_	429.23	429.50	26.14	9.5	*Stachybotrys* (plants, microscopic fungus) thrives on damaged cellulose-rich plant-based materials; it represents the secondary metabolites of *Stachybotrys* [44]

* Both compounds: as prepared and derivatized with TMS for GC-MS analysis. the siliconium ions (*m*/*z* 73), (*m*/*z* 147), (*m*/*z* 146), and (*m*/*z* 89) were generated independently from tetramethylsilane, hexamethyldisiloxane, hexamethyldisilazane, and methyltrimethylsilyl ether, respectively [45] and were all shown to form adducts with several neutral molecules such as phosphates and steroids containing electronegative centers.

Overall, microscopic analysis disclosed the presence of a non-saccate and inaperturate pollen with a star-like protoplast (Figure 6A). Under light microscopy the grain appears spherical (polar and equatorial axes both 31 µm) and is attributed to Cupressaceae-type pollen. Unfortunately, pollen from several species of Cupressaceae (e.g., *Juniper* sp. and *Cupressus* sp.) are considered morphologically uniform [46]; thus, it remains classified at family level. Thus, this evidence could testify to the presence of Cupressaceae in the area surrounding Cantagrilli and/or a possible employment of resinous material from these species [47], where pollen could have remained trapped. However, it is not possible to exclude the hypothesis that the pollen had been deposited randomly inside the adhesive mixture being transported by the wind or present in one of the ingredients used for its preparation (e.g., honeybee products).

The second class of composites identified in the sample are beeswax, as shown by the FTIR study. The presence of bee honey is excluded since two characteristic bands are present (1700–1703 cm^−1^), typical of aged and not fresh beeswax (which instead has the same bands recorded at higher frequencies: 1730–1736 cm^−1^). The FTIR results also show the presence of another component such as benzoyl resins that act as adhesive substances when thermally treated [48]. The benzoyl resin mainly contains benzoic derivatives (such as 4-hydroxybenzaldehyde, 3-hydroxybenzoic acid, 4-hydroxybenzoic acid, according to Table 3) and also vanillin [49] and bound vanillic acid [50], as reported in Table 3. A partially gelatinized starch granule was also observed in the pellet of the ancient sample. The polarizing filter confirmed that it belongs to this category of plant microparticles (Figure 6B). The granule was ovoid in shape (length 25.35 µm and width 19.18 µm); unfortunately, the hilum and lamellae were not visible, given the severe alteration of the internal crystalline structure. This change could be linked to immersion in heated water [51,52,53]. Its morphology fitted perfectly within the dimensional range of secondary starch produced by two groups of plant species: the Hordeeae Martinov tribe, to which the *Triticum* sp. L. and *Hordeum* sp. L. also belong, and *Quercus pubescens* Willd., according to [54] and the modern reference collection (Figure 6C and Figure 6D, respectively). Thus, the presence of this granule might indicate the use of starch glue, a water-soluble binding agent applied to stick together wood and metals [55]. Even in this case, as for pollen grain, it is not possible to exclude an accidental contamination event during the preparation of the adhesive material. Finally, in the sample, three pieces of microdebris of doubtful origin were found (Figure 6E,F). They appeared as fragments of chitinous exoskeleton equipped with a portion of abdomen with appendages, whose ends showed a branched structure. Because of the lack of unique diagnostic features, these microparticles have been generically attributed to microarthropods, maybe belonging to the Copepoda H. Milne-Edwards class. Since one of the components of starch glues is water [56], it is possible that the liquid used for the preparation of the archeological adhesive may have contained such aquatic microorganisms. Alongside this hypothesis, another one described in the literature [57,58,59,60] concerns the possibility of the microorganism’s inclusion into the resins/ambers of terrestrial plants (during their formation), by dissolution of calcium carbonate (especially, considering the karst nature of Cantagrilli).

## 4. Materials and Methods

### 4.1. Sampling

**Figure 7:**
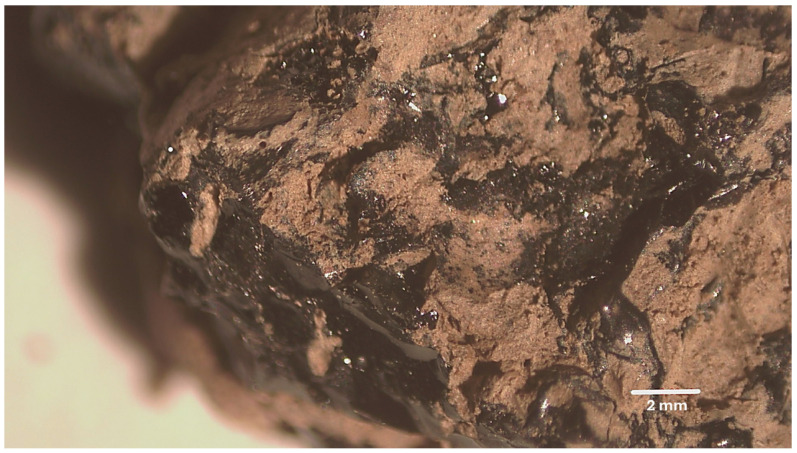
Optical microscopy micrograph of the analyzed sample partially covered by the excavation soil (metric reference 2 mm).

The sample analyzed comes from a fragment of black organic material, with a conchoidal fracture and resinous appearance, found in the anthropized layer together with stone tools and ceramic fragments (Figure 7).

### 4.2. Materials and Reagents

Phthalic anhydride, propionic acid, sodium hypochlorite, methanol, ethanol, dichloromethane (CH_2_Cl_2_), deuterated chloroform (CDCl_3_), chloroform (CHCl_3_), pyridine, bis (trimethylsilyl) trifluoroacetamide (BSTFA), trimethylchlorosilane, tetramethylsilane (TMS), KBr, and all reagents were purchased from (Sigma-Aldrich, Buchs, Switzerland); they were of analytical grade and used as received, without any purification step. A Milli-Q water system (Millipore, Burlington, MA, USA) was used to produce ultrapure water and all daily solutions; the latter were for analytical measurements.

The following GC-MS standards (pure substances for gas chromatographic analysis) were purchased from Merck (Darmstadt, Germany): tetramethylsilane (TMS, ≥99.0%), 3-(trimethylsilyl) propionic acid (≥99.9%), 3-methyl-4-(2,6,6-trimethyl-2-cyclohex-en-1-yl)-3-buten-2-one (≥99.8%), 12(*S*)-hydroxy-(5*Z*,8*E*,10*E*)-heptadecatrienoic acid (≥93.0%), 4-[[(8*R*,9*R*,10*R*,11*R*,13*S*,14*R*,17*S*)-17-acetyl-10,13-dimethyl-3-oxo 1,2,6,7,8,9,11,12,14,15,16,17-dodecahydrocy-clopen-ta[a]phenanthren-11-yl]oxy]-4-oxobutanoate (≥98.8%).

### 4.3. Procedures and Apparatuses

#### 4.3.1. Gas Chromatography–Mass Spectrometry (GC-MS) Analysis

Typically, 0.5–1.0 mg of material/sample was weighed and ground to a solid powder in an agate mortar. The experimental procedure performed to carry out the GC-MS analysis agrees with what has been reported in the literature [32,61], for very similar sample types. The collected powder was then extracted with dichloromethane (1 to 3 mL, HPLC grade) by ultrasonication (20 min, in an ultrasonic bath model LBS2-4,5 having a frequency of 50 KHz and working at R.T.) in a glass tube. An aliquot (100 µL) was submitted to trimethylsilylation in order to reduce the polarity of the samples and to increase their volatility.

After complete evaporation of the solvent, the trimethylsilyl (TMS) derivatives were obtained by adding 10 µL of CH_2_Cl_2_, 5 µL of pyridine, and 40 µL of BSTFA (bis(trimethylsilyl)trifluoroacetamide with 1% trimethylchlorosilane added).

After 20 min at R.T., the excess reagent and solvent were evaporated until dryness. The residue was dissolved in 10 to 100 µL of CH_2_Cl_2_ before analysis. An aliquot of 1 µL was injected into the GC apparatus. Combined GC-MS was carried out by using a Hewlett Packard 5972A mass selective detector (manufactured by Hewlett-Packard, Agilent Technologies, Palo Alto, CA, USA) equipped with an HP5890 Series II GC, having a split–spitless injector and used in splitless mode (at 340 °C with a 3 min purge time). Data were stored and processed using HPGI7701AA Chemstation software (Agilent-formerly HP-ChemStation software package, with specific versions like A.03.01). The analytical column used was a polyimide-clad 12 m × 0.22 mm I.D. fused silica capillary, coated with PB1 (SGE, Herts, UK) stationary phase (immobilized dimethyl polysiloxane, OV-1 equivalent, 0.1 µm film thickness). The GC temperature program was as follows: 2 min isothermal hold at 50 °C following injection and then 50–340 °C at a rate of 10 °C/min. The final temperature was held for 10 min.

Helium/He was used as the carrier gas at a constant flow of 1 mL/min and a column head pressure of 10 p.s.i. The transfer line temperature was set at 340 °C. Mass spectra were acquired by electron ionization (EI, 70 eV). Full-scan mass spectra over the range *m*/*z* 50–700. Peaks were identified by comparison with authentic standards of analytes (Sigma-Aldrich, Buchs, Switzerland) and the literature [20,62].

The GC-MS results provide an interesting overview concerning the chemical composition of the extract and highlight its richness in oxygenated and aromatic species, which are closely connected with the adhesive properties of these archeological samples, their uses, and the techniques applied to assemble them.

#### 4.3.2. Fourier-Transform Infrared Spectroscopy (FTIR) Analysis

The Fourier-Transform Infrared (FTIR) spectroscopy was performed in transmittance mode on samples previously assembled in KBr pellets using a Shimadzu Model Prestige 21 spectrophotometer (Kyoto, Japan).

#### 4.3.3. ^1^H-Proton Nuclear Magnetic Resonance Spectrometry (^1^H-NMR)

A few mg (~1.0 mg) of sample was dissolved in 750 µL of CDCl_3_ and placed in a 5 mm NMR tube. The sample showed good solubility in CDCl_3_, and there was no evidence of insoluble residues. ^1^H-NMR spectra were acquired on a Bruker-500 spectrometer apparatus (Billerica, MA, USA), operating at 500.13 MHz for the proton nucleus at R.T. The ^1^H-NMR spectra were obtained with the following acquisition parameters: 90° pulse width 10 ms, spectral width 12 ppm, and relaxation delay 2 s. The total number of scans for every experiment was 64 (4 dummy scans) and the acquisition time was set at 2.60 s. All ^1^H-NMR chemical shifts are relative to the peak of the solvent on the tetramethylsilane (TMS internal standard) scale.

#### 4.3.4. ^13^C-Carbon Nuclear Magnetic Resonance Spectrometry (^13^C-NMR)

^13^C-NMR spectra were obtained with the same instrument at 150 MHz in CDCl_3_. Deuterated chloroform (CDCl_3_) and TMS were used as solvent and internal standard, respectively. The ^13^C (150 MHz) spectra were recorded with a pulse duration of 30°, a recycle delay of 1.89 s, and 160 scans.

Chemical shifts are given in δ (ppm) values relative to those of the solvent signal CDCl_3_ on the tetramethylsilane (Sigma, St. Louis, MO, USA) scale.

#### 4.3.5. Archeobotanical Analysis

All decontamination and extraction procedures for microdebris were conducted in a cleanroom facility of the Laboratory of Archeobotany DAPHNE—Diet, Ancient DNA, Plant–Human Nexus, and Environment (Department of Biology—University of Rome “Tor Vergata”, Rome, Italy), not connected to modern botanical work and under strict environmental monitoring. To prevent any contamination, the rooms were used exclusively for the sample. and all the steps were conducted under a sterile vertical laminar flow hood. Thirty-two milligrams of sample were cleaned under stereomicroscope with a fine acupuncture needle to gently scrape off the debris attached to the external layer and immersed in ultrapure water and sodium hypochlorite (50:50; *v*/*v*) for 15 min. Then, the sample was washed three times with ultrapure water in order to remove any trace of contaminants. Once the surface was cleaned, the sample was dissolved in a solution of pure methanol for 4 days at 4 °C in the dark. After centrifugation at 13,000× *g* for 10 min, the supernatant was removed, and the pellet was subsequently mounted on microscope slides. Preceding the decontamination protocol, the sample was washed by sterile water and examined by optic microscope to confirm the efficacy of the method. Indeed, no microdebris was detected after sterilization.

The pellet was employed to evaluate the presence of microparticles by a ZEISS Axio Observer 7 optic microscope (Jena, Germany) equipped with polarized filters and Zen imaging software 2.6 operating at different magnifications. Palynomorph and starch elements and non-pollen palynomorphs (NPPs) were identified on morphometric features, grouped into preliminary types based on a shared morphology, and described using conventional nomenclature [63], literature works, reference collections of plant microparticles, and databases of wild and domestic plant species.

## 5. Conclusions

This study allowed us to combine chemical and archeobotanical information to better understand the composition of the archeological adhesive sample found in the archeological area of Cantagrilli (Florence). This type of multidisciplinary study was carried out for the first time at this sampling site (Cantagrilli, Florence) and for this kind of archeological adhesive.

In summary, the gas chromatographic analysis, coupled with all the other analytical techniques applied for the measurements, demonstrates the presence of triterpenes, aged beeswax (and not bee honey), benzoyl resin, and gelatinized starch. This multi-analytical approach allowed us to clarify the molecular nature of sample components as well as their state of denaturation and chemical decomposition/transformation.

These investigations could represent an important contribution to the understanding of prehistoric technologies and material processing.

## Figures and Tables

**Table 1:** The main molecular structures identified in the GC-MS chromatogram/mass spectrum for Retention Time (Rt) ≥ 12 (min).

No. of Identified Compounds	Compound Description	Retention Time (min)	*m*/*z* Range *
1	Triterpenes	≥22	400–550
2	Diterpenes	14–20	252–354
3	Hydrolysis products (Monoterpenoids and sesquiterpenes)	3–13	132–220
4	n-Alkane pattern (belonging to the long hydrophobic hydrocarbon chains—alkanes—of fatty acid esters)	3–13	73–118

* NIST library and mass spectra published in the literature for standard compounds.

**Table 2:** Main mass spectrum fragments; number of carbon and isoprene units in terpenes.

Hypothesized and Detected Compounds	MW	Main Fragments *m*/*z*	Class C Atoms No. of Isoprene Units (n)	Species	References
C_25_H_33_O_6_ (A)	429	[M]^+•^ molecular peak	Sesterterpenoids 25 5	Cupressaceae (e.g., *Juniper* sp. and *Cupressus* sp.)	[12]
C_17_H_25_^3^HO_3_ (C_17_H_28_O_3_) (B)	280	[M−149] where the fragment that is lost (149) is 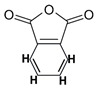 (C_8_H_3_^2^HO_3_)	Diterpenoids; sesquiterpenoids 17 3	Fatty acids, usually vegetable oils contained in alkyd resins	[17]
C_14_H_22_O (C)	206	[M−74] basic peak where the fragment that is lost (74) is propionic acid 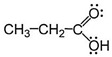	Monoterpenoids 14 2	Lower-molecular-weight fatty acids, C_6_ to C_14_	[16]

## Data Availability

Data are contained within the article.

## Supplementary Materials

The following supporting information can be downloaded at https://www.mdpi.com/article/10.3390/molecules31020274/s1: Scheme S1. Main identified compounds in the archeological sample. Scheme S2. The identified terpenoid compound C_25_H_33_O_6_ (*m*/*z* = 429), having the main fragmentation points. Scheme S3. Scheme of the steps involved in chemical fragmentation mechanisms, according to the mass spectrum highlighted in Figure 3 in the text. Table S1. Semi-quantitative concentrations of identified compounds by targeted-standard GC-MS analysis. Text S1. FTIR molecular adsorption band assignments for the sample. Text S2. ^1^H-NMR and ^13^C-NMR spectral data and chemical shift assignments.
