# Supplementary material for: Various Analytical Techniques Reveal the Presence of Damaged Organic Remains in a Neolithic Adhesive Collected During Archeological Excavations in Cantagrilli (Florence Area, Italy)
Source: Molecules. 2026 Jan 13;31(2):274. doi: 10.3390/molecules31020274 (PMC12844484; doi:10.3390/molecules31020274)
Supplement: Supplementary file 1 [file molecules-31-00274-s001.zip › molecules-4038860-supplementary.pdf]

molecules

MDPI

Article

Various Analytical Techniques Reveal the Presence of Damaged Organic Remains in a Neolithic Adhesive Collected During Archeological Excavations in Cantagrilli (Florence Area, Italy)

Federica Valentini [1,*], Lucia Sarti [2], Fabio Martini [3], Pasquino Pallecchi [3], Ivo Allegrini [4], Irene Angela Colasanti [1,5], Camilla Zaratti [1,5], Andrea Macchia [5], Angelo Gismondi [6], Alessia D'Agostino [6], Antonella Canini [6] and Anna Neri [7]

[1] Sciences and Chemical Technologies Department, Tor Vergata University, Via della Ricerca Scientifica 1, 00133 Rome, Italy; ireneangela.colasanti@students.uniroma2.eu (I.A.C.); camilla.zaratti@students.uniroma2.eu (C.Z.)
[2] Dipartimento Scienze Storiche e Beni Culturali, University of Siena, Via Roma 53, 53100 Siena, Italy; lucia.sarti@unisi.it
[3] Museo e Istituto Fiorentino di Preistoria, Via dell'oriolo 24, 50122 Firenze, Italy; fabio.martini@unifi.it (F.M.); p.pallecchi@museofiorentinopreistoria.it (P.P.)
[4] Envint Srl, Via Paradiso 65 a, Montopoli di Sabina, 02034 Rieti, Italy; ivo.allegrini@tiscali.it
[5] YOCOCU APS, Via Torquato Tasso 108, 00185 Roma, Italy; aps@yococu.com
[6] Laboratory of Archaeobotany DAPHNE (Diet, Ancient DNA, Plant-Human Nexus, and Environment), Department of Biology, Tor Vergata University, Via della Ricerca Scientifica 1, 00133 Rome, Italy; gismondi@scienze.uniroma2.it (A.G.); d.agostino@scienze.uniroma2.it (A.D.); canini@uniroma2.it (A.C.)
[7] Department of Biomedicine and Prevention, Tor Vergata University, Viale Montpellier 1, 00133 Rome, Italy; anna.neri@uniroma2.it
* Correspondence: federica.valentini@uniroma2.it:

Supplementary Material

 https://doi.org/10.3390/molecules31020274

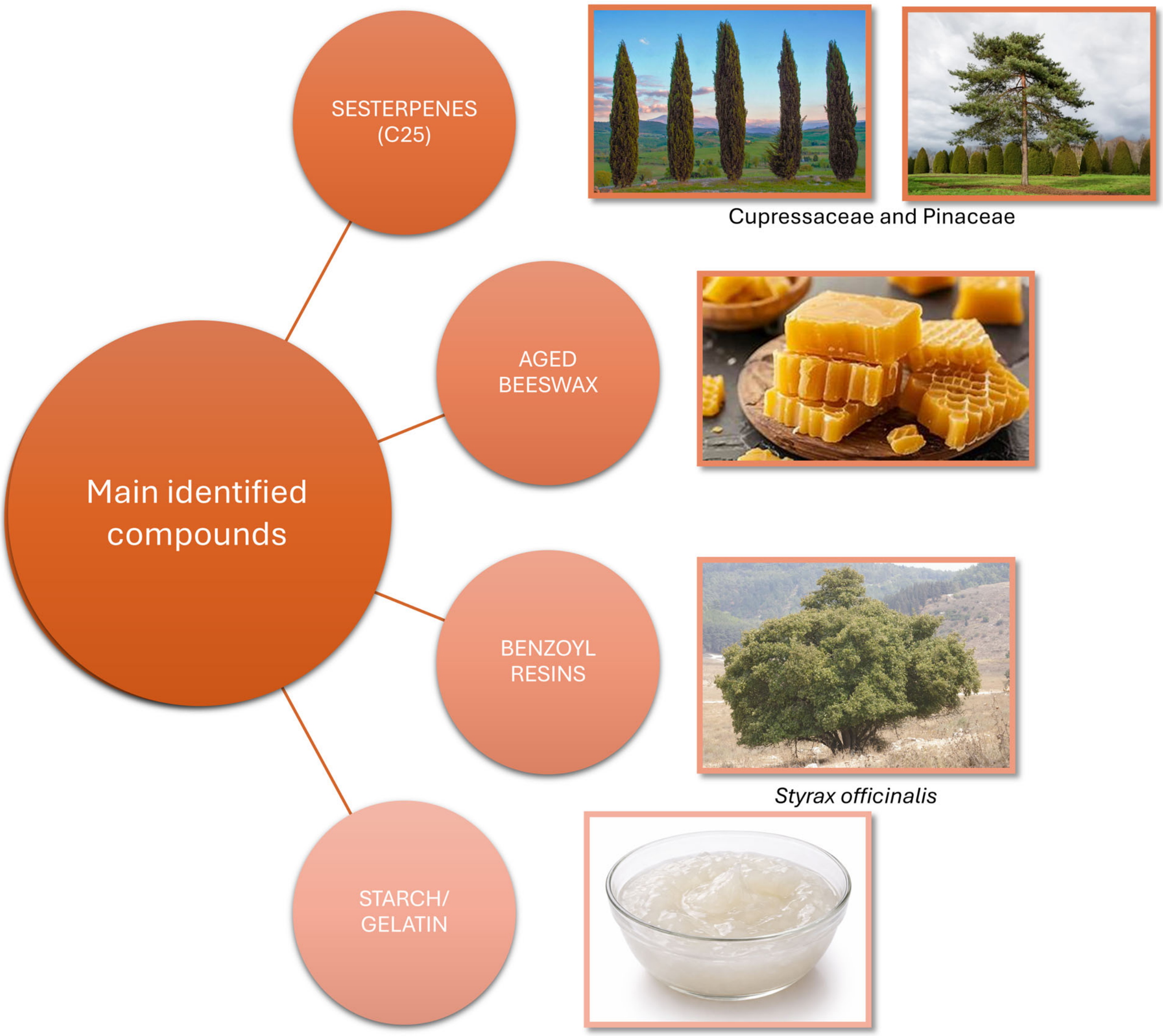

Scheme S1. Main identified compounds in the archaeological sample. The only source of benzoyl resin in the Mediterranean area is *Styrax officinalis*, [Modugno, F.; Ribechini, E.; Colombini, M. P. Aromatic resin characterization by gas chromatography–mass spectrometry. *J. Chromatogr. A* **2006**, 1134, 298–304. https://doi.org/10.1016/j.chroma.2006.09.010].

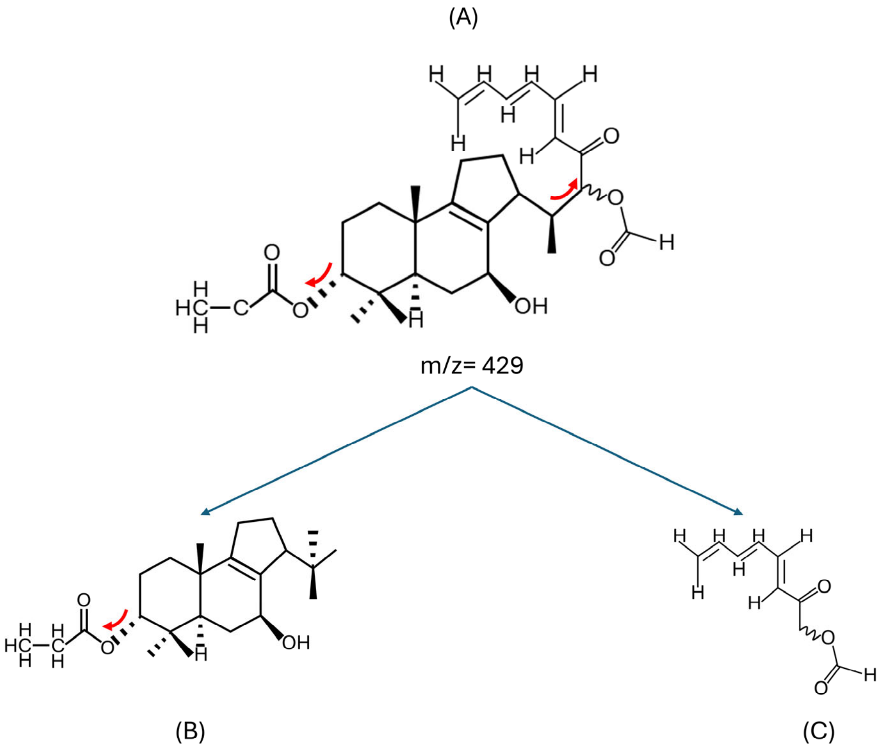

Scheme S2. The identified terpenoid compound: $C_{25}H_{33}O_6$ (m/z = 429), having the main fragmentation points.

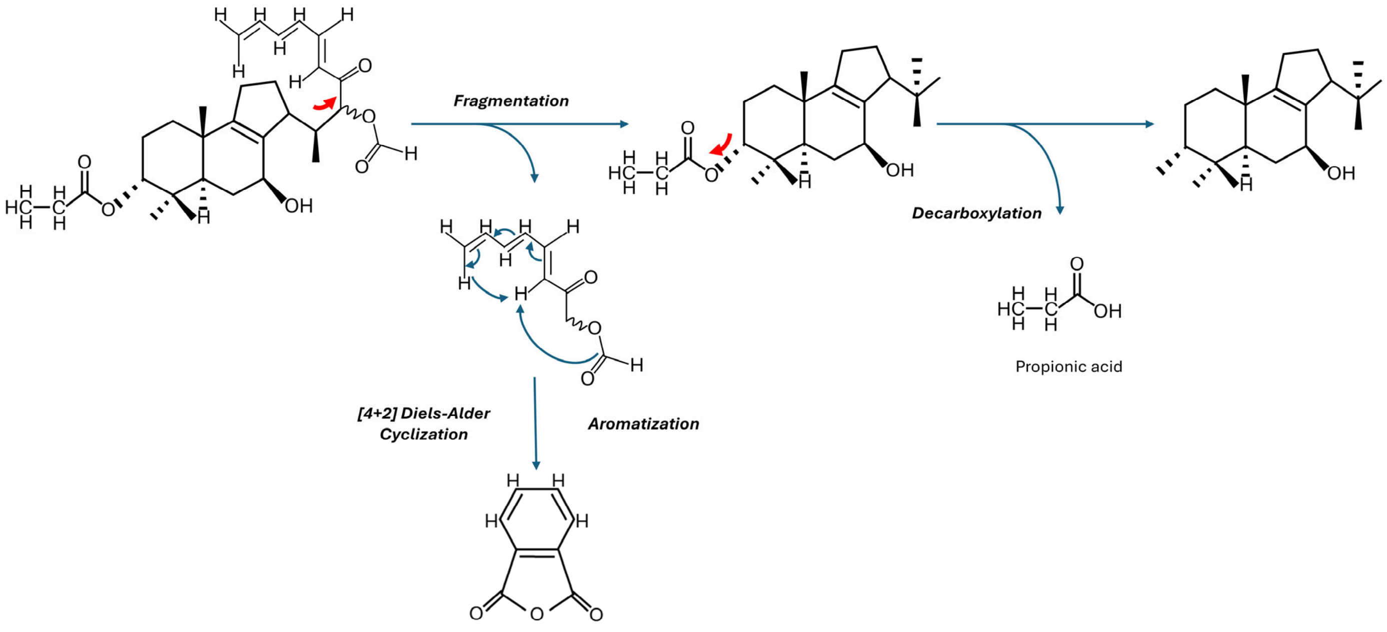

Scheme S3. Scheme of the steps involved in chemical fragmentation mechanisms, according to the Mass spectrum highlighted on Figure 3 in the text. This may explain the damage processes associated with exposure to light, air, and environmental microorganisms.

https://doi.org/10.3390/molecules31020274

Table S1. Semi-quantitative concentrations of identified compounds by targeted-standard GC-MS analysis.

Compound	Concentration [ng/mL]
3-Methyl-4-(2,6,6-trimethyl-2-cyclohex-en-1-yl)-3-buten-2-one	6383
12(S)-Hydroxy-(5Z,8E,10E)-heptadecatrienoic acid	2044
4-[[(8R,9R,10R,11R,13S,14R,17S)-17-acetyl-10,13-dimethyl-3 oxo1,2,6,7,8,9,11,12,14,15,16,17-dodecahydrocyclopenta[a]phenanthren-11-yl]oxy]-4-oxobutanoate	64

Supporting Information Text

Text S1. FTIR molecular adsorption band assignments for the sample.

The Fourier Transform Infrared (FTIR) spectroscopy was performed in transmittance mode on samples, previously assembled in KBr pellets using a Shimadzu Model Prestige 21 spectrophotometer. This latter offers comprehensive functionality for spectral data processing, molecular bands identification, and qualitative analysis. It is a network-enabled software featuring spectral resolution, bands fitting algorithms, and spectral libraries, enhancing efficiency in FTIR research and analysis in Cultural heritage field.

Text S2. ^{1}H-NMR and ^{13}C-NMR spectral data and chemical shift assignments.

^{1}H-NMR and ^{13}C-NMR spectra were acquired on a Bruker-500 spectrometer apparatus, which is equipped by a new NMR-based Advanced Chemical Profiling Software, suitable to offer a truly automated, end-to-end workflow from sample to actionable information, eliminating the need for hands-on spectroscopist management.

The processing of the results and the comparison with a very rich database allows a truly exhaustive and complete interpretation of the results. It is easily applicable to chemical compounds (as deposited, pristine raw materials), in pure form or mixtures allowing identification and quantification of each pristine single components, process reaction intermediates, and final product formulations.

https://doi.org/10.3390/molecules31020274
